# Use of medicinal plants as feed additives in the diets of Mozambique tilapia (*Oreochromis mossambicus*) and the African Sharptooth catfish (*Clarias gariepinus*) in Southern Africa

**DOI:** 10.3389/fvets.2022.1072369

**Published:** 2022-12-16

**Authors:** Esau Matthews Mbokane, Ngonidzashe Adreck Gukuta Moyo

**Affiliations:** ^1^Aquaculture Research and Development, Department of Fisheries, Forestry and the Environment, Cape Town, South Africa; ^2^Aquaculture Research Unit, School of Agricultural and Environmental Sciences, University of Limpopo, Mankweng, South Africa

**Keywords:** freshwater aquaculture, immunity, disease resistance, growth promoters, reproduction

## Abstract

Mozambique tilapia (*Oreochromis mossambicus*) and the African Sharptooth catfish (*Clarias gariepinus*) are the most farmed freshwater fish species in Southern Africa. However, production in the freshwater aquaculture sector has remained low due to, among other key factors, high cost of feeds, disease outbreaks, and poor sexual development in broodstock. Small-scale farmers are affected the most because they often lack resources and cannot afford expensive commercial diets, antimicrobials, and synthetic hormones needed to regulate reproduction. Among the proposed solutions, the inclusion of medicinal plants as feed additives is the most promising alternative to enhance growth performance, disease resistance and reproduction in fish. Plants contain various compounds such as polyphenols, carbohydrates, amino acids, flavonoids, alkaloids, tannins, organic acids, volatile oils, polysaccharides, minerals, and vitamins, some of which are necessary for growth and improving immunity or overall wellbeing in fish and other animals. However, the utilization of plants as feed additives in aquafeeds is still limited in Southern Africa. This paper reviews the potential role that medicinal plants can play as feed additives in order to promote growth performance, immunity, disease resistance, and reproduction in the culture of *O*. *mossambicus* and *C*. *gariepinus* in Southern Africa. The objective was to consolidate information about plants that can be specifically applied in freshwater aquaculture in Southern Africa by highlighting their availability and efficacy as either growth promoters or immunostimulants or fertility enhancer.

## Introduction

Freshwater aquaculture is one of the most important and fastest-growing food-producing industries worldwide. In developing countries, freshwater aquaculture plays an important role as an alternative source of cheap animal protein. It is also considered an important sector that can create job opportunities for poor communities. In Southern Africa, for example, most governments consider the development of freshwater aquaculture as one of the potential solutions to reduce poverty. Many countries in the region (i.e., Malawi, Zambia, South Africa, Botswana, Eswatini, Namibia, Mozambique, and Zimbabwe) have developed action plans and policies focusing on reducing hunger and creating employment opportunities by increasing freshwater aquaculture production (1). The widely farmed freshwater fish species in Southern Africa are Mozambique tilapia (*Oreochromis mossambicus*) and the African Sharptooth catfish (*Clarias gariepinus*). In Southern Africa, the culture of both species is mainly dominated by the small-scale sector, which is considered an important component of inland aquaculture in many countries. However, production of these fish species in the small-scale sector in the majority of countries in Southern Africa has remained low when compared to other countries on the African continent (e.g., Egypt, Nigeria, Uganda, Ghana, and Tunisia).

Lack of good quality feeds, disease outbreaks, and poor quality of broodstock have been identified as among the key factors hindering production and profitability of the culture of *O*. *mossambicus* and *C*. *gariepinus* in Southern Africa. Moyo and Rapatsa (1) reviewed several factors affecting tilapia production in Southern Africa and they found that, among others, availability of quality feeds, diseases, and poor quality of fingerlings were the most important factors affecting tilapia production. Most farmers in Southern Africa rely on commercial feeds for their operations. However, some countries do not manufacture their feed, but import from other countries, which makes the price of fish feeds more expensive in those countries. Even in countries such as South Africa, Zambia, and Zimbabwe where there is several fish feed producers; commercial feeds are becoming increasingly unaffordable due to the over-reliance on expensive feed ingredients such as fishmeal. This renders most small-scale enterprises unsustainable as farmers often lack resources and cannot afford commercial diets. As a result, farmers resort to using poor-quality feeds, thus compromising growth of the fish.

Disease outbreaks are also among the major constraints affecting aquaculture production in Southern Africa. It has been reported that the outbreak of diseases in tilapia and catfish farms is one of the most important factors affecting production and profitability (2, 3). In Southern Africa, tilapia and catfish are mainly affected by bacterial, fungal, parasitic, and viral diseases (4). Common pathogens such as *Aeromonas hydrophila* and *Saprolegnia parasitica* have proven elusive to eliminate in the small-scale sector in Southern Africa (1, 4). These are considered opportunistic pathogens because they target immune-compromised fish. Culture conditions such as high stocking density, handling, and poor water quality create stress in captive fish, which compromises the immune capacity in fish and make them susceptible to opportunistic pathogens (5). In Southern Africa, *O*. *mossambicus* and *C*. *gariepinus* are mainly cultured in earthen ponds because this is the easiest way to minimize feeding costs due to the abundance of natural food in ponds. However, fish cultured in outdoor ponds are exposed to stressful conditions due to poor water quality and low water temperatures in winter, which reduce their immune response. This increases their susceptibility to opportunistic pathogens. Poor nutrition (as mentioned some farmers use poor-quality feeds) also affects the health of fish and increases their susceptibility to infectious agents. Management of diseases in the small-scale sector is a major challenge; primarily because farmers do not implement strict biosecurity protocols intended to minimize the risks of spreading infectious agents on fish farms. Farmers also do not have access to antibiotics and synthetic chemicals because they tend to be expensive. Furthermore, it is widely known now that the use of antibiotics and synthetic chemicals in aquaculture has been restricted due to their negative impact on fish and consumer health (6).

Another challenge affecting production in the small-scale sector in Southern Africa is the availability of good quality fingerlings. Moyo and Rapatsa (1) noted that precocious breeding and fingerling quality in tilapia was seriously affecting tilapia production in Southern Africa. In catfish production, farmers are also struggling with the availability of high-quality seeds. This is exacerbated by the fact that the African Sharptooth catfish do not breed in captivity without artificial inducement. This means that sufficient skills and training are needed to condition the fish properly to reach gonadal maturation at the appropriate time. However, farmers often struggle to properly condition their broodstock in captivity due to the seasonal natural breeding cycle in *C*. *gariepinus*, which normally starts with the onset of the rainy season in September in Southern Africa. Therefore, there is a need for freshwater fish farmers to find alternative sources of feed ingredients that can be used to replace expensive ingredients such as fishmeal or feed additives that can be used to promote growth performance, disease resistance, and reproduction in *O*. *mossambicus* and *C*. *gariepinus*.

One of the cheapest and promising alternative sources of feed additives include the use of medicinal plants. Plants contain various bioactive compounds such as terpenoids, alkaloids, flavonoids, tannins, saponins, pigments, phenolics, terpenoids, steroids, and essential oils (7, 8). A number of these compounds found in most medicinal plants possess various pharmacological functions such as antistress, appetite stimulation, growth promotion, immunostimulation, aphrodisiac, antimicrobial, antifungal, and antiparasitic properties (7). This shows that plants can be used as feed additives or substitutes for conventional feed ingredients in aquafeeds fish. Several studies have shown that plants can boost growth performance and disease resistance in fish (7–10). Some plants have been reported to enhance gonadal development in some fish species (11). However, information regarding the use of plants in aquaculture in Southern Africa is very limited. Given the large number of plants that have been investigated for their possible inclusion in fish feeds, there is a need to review the literature in order to identify plants that can potentially be used as feed additives in the diet of *O*. *mossambicus* and *C*. *gariepinus* in Southern Africa. As far as it could be established, there are few published reviews on the use of plants in tilapia production (12, 13). However, these reviews have mainly focused on the application of plants in tilapia species as a group and not specifically on *O*. *mossambicus*. On the other hand, no reviews have specifically focused on the use of plants in the culture of *C*. *gariepinus*. Generally, the available reviews on the use of plants in aquaculture published over the last few years have focused on the broad application of plants in different marine and freshwater fish species of aquaculture importance (7–10). Therefore, the present paper focuses on the potential application of medicinal plants in *O*. *mossambicus* and *C*. *gariepinus* in Southern Africa. The specific objective was to highlight indigenous and common plants that can be used to improve growth performance, disease resistance and reproduction in these fish species in Southern Africa. This paper also proposes some interventions needed to encourage the utilization of medicinal plants in aquafeeds in the small-scale aquaculture sector in Southern Africa.

## Use of plants as growth promoters in *Oreochromis mossambicus* and *Clarias gariepinus*

Medicinal plants are considered suitable feed additives in aquafeeds because some of them contain adequate amounts of proteins, vitamins, essential amino acids, unsaturated fatty acids, and minerals that can meet the nutritional requirements for some of the widely cultured fish species (14). Others have been reported to contain bioactive compounds that can enhance the activities of digestive enzymes (15). Pu et al. (16) stated that medicinal plants could improve growth performance in fish due to the positive effect some bioactive substances have on metabolism and their capacity to enhance protein synthesis and activate digestive enzymes. Several plants have been investigated for their growth-promoting effect in *O*. *mossambicus* and *C*. *gariepinus* (Table 1). These plants have been shown to improve growth performance (weight gain, specific growth rate, feed conversion ratio, and feed intake) in *O*. *mossambicus* and *C*. *gariepinus* (Table 1). It is of interest that most of these plants have a wide distribution in Southern Africa, where they are widely used in traditional medicine, food (vegetables) and as spices, which make them to have an enormous potential to be used in freshwater aquaculture. For instance, Hlophe-Ginindza and Moyo (17) demonstrated that the total replacement of fishmeal with *Cenchrus clandestinus* (kikuyu grass) leaf meal in the diets of *O*. *mossambicus* improved growth performance, protein digestibility, and feed utilization. Similarly, Hlophe-Ginindza and Moyo (18) found that the kikuyu-based leaf meal resulted in significantly higher growth performance, feed, and protein utilization in *C*. *gariepinus*. Kikuyu grass is a very common warm-season grass in Southern Africa and it is known for its rapid growth. It has a high protein content and good amino acid profile (19, 20), which shows that it could be used as a protein source in fish diets. However, its potential use in aquaculture in Southern Africa has not been widely publicized. It is suggested that more studies on the use of kikuyu in these fish species be carried out at farm level because of its high potential as a replacement for fishmeal.

**Table 1:** Plants used to promote growth performance in *Oreochromis mossambicus* and *Clarias gariepinus*.

**Fish species**	**Plant species**	**Dose**	**Effect**	**References**
*Oreochromis mossambicus*	*Aegle marmelos*	1%	↑ Specific growth rate	(23)
	*Cynodon dactylon*	1%	↑ Specific growth rate	(23)
	*Withania somnifera* (ashwagandha or winter cherry)	1%	↑ Specific growth rate	(23)
	*Zingiber officinale* (ginger)	1%	↑ Specific growth rate	(23)
	*Zingiber officinale*	1 and 2%	↑ Growth performance (weight gain, and specific growth rate	(24)
	*Zingiber officinale*	20%	↑ Weight gain, specific growth rate, and protein efficiency ratio	(25)
	*Cuminum cyminum* (cumin)	0, 0.5, 1.0, 1.5, and 2.0%	Weight gain, specific growth rate, and feed conversion ration	(37)
	*Tribulus terrestris*	0, 200, 400, 600, and 800 mg kg^−1^	↑ Final weight, weight gain, and specific growth rate	(40)
	*Astaxanthin, paprika*, and *capsicum*	40 and 60 mg kg^−1^	↑ Weight gain, specific growth rate, and food conversion ratio	(47)
	*Pimenta dioica* (Allspice)	0, 5, 10, 15, and 20 g kg^−1^	↑ Growth performance and feed utilization	(45)
	*Cenchrus clandestinus* (kikuyu grass)	0, 25, 50, 75, and 100% (fishmeal replacement)	↑ Growth performance, protein digestibility, and feed utilization	(18)
*Clarias gariepinus*	*Ocimum gratissimum* (clove basil)	0.0, 5, 10, and 15 g kg^−1^	↑ Weight gain, specific growth rate, feed conversion ratio, and feed intake, villi length, villi width, and absorption area	(26)
	*Allium sativum* (garlic)	0 and 5 g kg^−1^	↑ Weight gain and specific growth rate	(27)
	*Allium sativum*	0, 0.5, 1.0, 2.0, and 4.0%	↑ Feed intake, feed conversion ratio, and protein efficiency ratio	(28)
	*Aloe vera*	0.5, 1.0, 2.0, and 4.0%	↑ Specific growth rate, weight gain, absolute growth rate, and protein efficiency ratio	(30)
	*Rosmarinus officinalis*	0, 0.25, and 0.5%	↑ Weight gain, specific growth rate, and best food conversion rate	(34)
	*Sonneratia caseolaris* (apple mangrove)	1.59, 2.22, and 3.17 mg ml^−1^	↑ Weight gain and specific growth rate	(35)
	*Garcinia kola* (bitter kola)	0, 50, 100, 150, 200, and 250g kg^−1^	↑ Weight gain, specific growth rate, feed conversion ratio, and protein efficiency ration	(38)
	*Garcinia kola*	0 and 1.0 g kg^−1^	↑ Weight gain and specific growth rate	(39)
	*Mitracarpus scaber*	0, 2, 4, and 6 g kg^−1^	↑ Weight gain, specific growth rate, feed conversion ratio, and feed intake	(41)
	*Telfairia occidentalis* (fluted pumpkin)	5, 10, 15, and 20 g kg^−1^	↑ Weight gain, specific growth rate, feed conversion ratio, and feed intake	(42)
	*Telfairia occidentalis*	0, 0.5, 1.0, 1.5, and 2.0%	↑ Weight gain	(43)

The symbol ↑ represents an increase in all parameters.

Feeding trials have also been carried out using *Moringa oleifera* (moringa) leaf meal as a substitute for fishmeal in *O*. *mossambicus* and *C*. *gariepinus* diets (17, 18). Unlike the kikuyu-based diets, Hlophe-Ginindza and Moyo (17, 18) found that the total replacement of fishmeal with *M*. *oleifera* compromised growth performance in both *O*. *mossambicus* and *C*. *gariepinus*. They concluded that *M*. *oleifera* was not a suitable replacement for fishmeal in the diets of these fish species. It, however, appears that the dosages used by Hlophe-Ginindza and Moyo (17, 18) were excessively high than those commonly reported in the literature. For example, these authors replaced fishmeal up to 100%. Fishmeal makes up a significant portion in aquafeeds and replacing it completely with a plant material might have significantly increased the presence of indigestible material [anti-nutritional factors (ANFs)] in the diet, thus compromising growth performance. Hlophe-Ginindza and Moyo (18) observed that *M*. *oleifera* had high levels of ANFs such as phytic acid, saponins, and tannins compared to kikuyu. Anti-nutritional factors (e.g., tannin, phenol, saponin, lignin, and alkaloid) bind to digestive enzymes and reduce digestibility and nutrient availability (21). Some plants possess high levels of protease inhibitors such as lectins, phytic acid, saponins, phytoestrogens, antivitamins, allergens, tannins, gossypol, and glucosinolates (21), which may lead to nutrient deprivation and poor growth performance in fish. Excessive amounts of ANFs found in some plants can also affect the gut morphology and population of probiotics. Hlophe-Ginindza and Moyo (18) found that the fish fed with the moringa-based diets had intestinal and liver alterations when compared to fish fed with the kikuyu-based diets. However, Hlophe-Ginindza and Moyo (22) showed that the levels of ANFs in plants can be reduced by the use of exogenous enzymes, which will increase the availability of nutrients. For example, Hlophe-Ginindza and Moyo (22) showed that the inclusion of a commercial multi-enzyme (Natuzyme50^®^) in the diets of *O*. *mossambicus* in which fishmeal had been substituted with kikuyu reduced the levels of ANFs and improved feed utilization and growth performance. Natuzyme50^®^ is a combination of different enzymes such as amylase, acid phytase, pentosanase, hemicellulase, cellulase, protease, xylanase, b-glucanase, lipase, pectinase, amyloglucosidase, and acid phosphatase. This is an indication that the nutritional profile of some plants can be enhanced using exogenous enzymes to make them safe feed additives. *Moringa oleifera* is widely available and it has been associated with many health benefits in humans and, as a result, many people in Southern Africa have been encouraged to cultivate it. The leaves of *M*. *oleifera* possess high protein content, ascorbic acid, carotenoids, and iron (18), which make it a candidate as a feed additive in aquafeeds. Nevertheless, there is still a need for further investigations to look at the use of exogenous enzymes in moringa supplementation diets in both *O*. *mossambicus* and *C*. *gariepinus*.

The use of extracts from plants such as *Aegle marmelos, Cynodon dactylon, Withania somnifera* (ashwagandha or winter cherry), and *Zingiber officinale* (ginger) improved feed efficiency and growth performance in *O*. *mossambicus* (23). Oparaku et al. (24) also found that *Z*. *officinale* powder improved growth performance in *C*. *gariepinus*. Similarly, Olaniyi et al. (25) reported that dietary supplementation with *Z*. *officinale* powder at 20% led to significant increases in weight gain, specific growth rate, and protein efficiency ratio in the same fish species. These studies show that ginger contains compounds that that are beneficial for growth in the fish. Ginger is one of the most common plants in Southern Africa that has an enormous potential to be used as a feed additive in aquaculture. It is readily available and many households use it for culinary and medicinal purposes. In addition, many studies have shown that it can enhance growth in other fish species (9).

Another plant of interest in freshwater fish farming is *Ocimum gratissimum* (clove basil). Abdel-Tawwab et al. (26) reported that growth performance and feed intake were significantly improved in *C*. *gariepinus* fed with diets enriched with *O*. *gratissimum* leaf extract. *Ocimum gratissimum* is a plant native to Southern Asia, Africa, and Madagascar, although it has been naturalized in many countries (West Indies, Polynesia, Mexico, Bolivia, Panama, Brazil, and Hawaii). In Africa, *O*. *gratissimum* is considered a common culinary herb and can be easily accessed by many fish farmers. *Allium sativum* (garlic) is also one of the most common medicinal plants with a significant potential application in freshwater aquaculture in Southern Africa. It is associated with many health benefits in humans (for many years it has been used for medicinal and culinary purposes). *Allium sativum* is known to possess compounds with various functions such as growth-promoting properties. Tiamiyu et al. (27) demonstrated that weight gain increased significantly in *C*. *gariepinus* fed with diets enriched with *A*. *sativum* powder. Gabriel et al. (28) also observed that *A*. *sativum* polysaccharide crude extract increased feed utilization and growth performance in *C*. *gariepinus*. Several studies have also reported that the incorporation of *A*. *sativum* in aquafeeds improved growth in various fish species (7, 9, 10). This suggests that farmers in the small-scale sector can benefit from using this plant due to its availability in many regions.

*Aloe vera* (true aloe) is another plant that has been reported to have health-boosting properties and has a long history of being used in traditional medicine (29). Gabriel et al. (30) found that feeding *C*. *gariepinus* fingerlings with diets supplemented with 0.5, 1.0, 2.0, and 4.0% *A. vera* polysaccharide crude extracts significantly increased growth performance (specific growth rate, weight gain, absolute growth rate) and protein efficiency ratio. The authors concluded that dietary supplementation with *A*. *vera* polysaccharide crude extracts could be suitable for growth and feed utilization in *C*. *gariepinus*. *Aloe vera* is a common medicinal plant that grows widely in both tropical and subtropical regions. It is known to possess a range of bioactive compounds, polysaccharides, amino acids, vitamins (A, B1, B2, B6, C, E, and folic acid), minerals (Ca, Mg, and Na), enzymes (lipase, amylase, carboxypeptidase), anthraquinones, salicylic acid, lignin, saponins, fatty acids, and hormones (31, 32). The presence of these compounds indicates that *A*. *vera* can be a suitable feed additive in aquafeeds for catfish. It has also been shown to enhance growth performance in tilapia species (GIFT) (33). Although the focus of this study is not on GIFT, the results from Gabriel et al. (33) suggest that *A*. *vera* could also be used to improve growth performance in *O*. *mossambicus* because the nutritional requirements for tilapia species are similar.

The use of *Rosemaria officinalis* (rosemary) extracts in the diets of *C*. *gariepinus* resulted in higher weight gain, specific growth rate, and best food conversion rate (34). The plant has also been shown to enhance growth performance in other fish species (7). *Rosmarinus officinalis* is widely used in traditional medicine for a range of human ailments such as colds, headaches, stomach disorders, inflammation, and heart disease (34). Native to the Mediterranean region, *R*. *officinalis* is now cultivated around the world. Fish farmers in Southern Africa can use this plant as a feed additive in aquafeeds due to its availability and diverse phytochemical profile. *Sonneratia caseolaris* (apple mangrove) leaf extract is another common plant that enhanced growth performance in *C*. *gariepinus* (35). This plant is mainly used for the commercial production of apple fruits. Although its leaves may not be easily accessible by all communities, some households in Southern Africa grow it on their backyards. Knowledge of its potential use in fish farming can thus benefit some of the small-scale farmers. *Cuminum cyminum* (cumin) is another plant that can be considered for inclusion in aquafeeds. This plant is used as a spice and it is thought to possess therapeutic properties (36). It has since been introduced to many countries around the world (36). Since cumin is often used in bird food and has been introduced in many regions, many fish farmers can easily access it or grow it on their properties. Yilmaz et al. (37) showed that the use of cumin seed meal as a feed additive in the diets of *O*. *mossambicus* improved growth performance (weight gain, specific growth rate, and feed conversion ration).

The other plant with a potential to be used as a feed additive in the diets of fish is *Garcinia kola* (bitter kola). This plant possess phytochemicals that have been reported to have various health benefits and it has been reported to enhance growth performance in fish (38). Results from Olaniyi et al. (38) showed that feeds supplemented with *G*. *kola* seed meal promoted growth performance in *C*. *gariepinus*. Dada and Ikuerowo (39) also showed that diets supplemented with extracts of *G*. *kola* resulted in higher weight gain and specific growth rate in the same fish species. However, its limitation for its application in aquaculture in Southern Africa is that it is not widely distributed in the region. *Garcinia kola* occurs abundantly in countries such as Democratic Republic of the Congo, Sierra Leone, Ivory Coast, Mali, Ghana, Liberia, Cameroon, The Gambia, Benin, Senegal, Gabon, and Nigeria. It is a plant that has been used for many centuries by traditional healers to treat minor illnesses such as coughs and fever (38). The inclusion of *Tribulus terrestris* extracts in *O*. *mossambicus* diets increased growth performance (40). Native to the Mediterranean region, *T*. *terrestris* is now widely distributed around the world. However, it is considered invasive in other countries and thus its potential application in aquaculture is very limited. Adeshina et al. (41) found that the inclusion of *Mitracarpus scaber* leaf extract in the diets of *C*. *gariepinus* resulted in better growth performance. *Mitracarpus scaber* is commonly employed in traditional medicine in West Africa to treat various human ailments (i.e., leprosy, hepatic diseases, dyspepsia, headaches, toothache, and amenorrhoea venereal diseases) (41).

Dada (42) also showed that diets supplemented with *Telfairia occidentalis* (fluted pumpkin) leaf meal improved growth and feed utilization in *C*. *gariepinus* fingerlings. Dada's (42) findings are in agreement with Idowu et al. (43) who also demonstrated that feeding *C*. *gariepinus* diets supplemented with *T*. *occidentalis* leaf meal resulted in significantly higher weight gain compared to the control. These authors concluded that *T*. *occidentalis* leaf meal could be used as a plant additive in the diets of *C*. *gariepinus*. *Telfairia occidentalis* is a green leafy vegetable native to Southern Nigeria where it is mainly used as a vegetable and in herbal medicine. Its leaves are rich in protein, oil, vitamins (A, C, and K), minerals (calcium, zinc, potassium, cobalt, copper, iron), and low in crude fiber (44), making it one of the plants most suitable as a feed additive in aquafeeds. However, its distribution outside its native territory is limited, thus limiting its use in aquaculture in other regions such as Southern Africa. Yilmaz and Ergün (45) reported that the application of *Pimenta dioica* (allspice) powder as a feed additive in *O*. *mossambicus* diets improved growth performance (weight gain). This study concluded that allspice could be used as a growth promoter to improve feed utilization in *O*. *mossambicus*. Allspice is native to the West Indies and it is commonly used as a spice (46). It has now been introduced to many countries in tropical regions mainly as an ornamental species and for its spice known as allspice (46). Although allspice is considered highly invasive and may not be allowed in other countries, the fact that it is a popular spice shows that fish farmers with access to it can use it in fish.

It has also been shown that compounds such as carotenoids abundantly found in some commonly grown plants can be used as feed additives in aquafeeds to improve growth performance in fish. Yilmaz et al. (47) found that the addition of astaxanthin, paprika, and capsicum (40 and 60 mg/kg) improved weight gain, specific growth rate, and food conversion ratio in *O*. *mossambicus* compared to the control fish. They concluded that the carotenoid supplemented diets could be used as an alternative natural source of carotenoid in the diets of *O*. *mossambicus* in order to promote growth performance. Many widely grown and consumed plants such as *Capsicum annuum* (red peppers) are rich in carotenoids, which shows that many fish farmers can benefit from using some of these widely consumed plants to enhance growth in fish. With regards to the effect of plants on growth performance in fish, it is clear that there many plants with the potential to improve growth performance in *O*. *mossambicus* and *C*. *gariepinus*. Further studies should be undertaken to determine their successful application on fish farms.

## Effect of plants on immunity and disease resistance in *Oreochromis mossambicus* and *Clarias gariepinus*

One of the best ways to prevent diseases on fish farms is to use feed additives that can strengthen the immune system of the fish. Farmers in Southern Africa can benefit from the use of plant-based products as feed additives because some of the compounds found in medicinal plants are reported to possess immunostimulatory activities (10, 48). Strengthening of the immune system of the fish appears to be a cheaper strategy to control diseases because the enhanced immune response of the fish would be able to resist infections, significantly reducing resources spent on treating sick fish. Bioactive compounds from plants have been shown to increase the activities of cells and molecules involved in innate immune response (non-specific) in fish (48, 49). Components of the innate immune systems include white blood cells such as neutrophils, monocytes, macrophages, granulocytes, and humoral elements like lysozyme (48). These components are considered the first line of defense against pathogenic agents (bacteria, fungi, parasites, and viruses). They are the first cells to come into contact with invading pathogens and protect against a variety of pathogens (48). In addition, plants have been reported to contain bioactive compounds that act as antibacterial, antifungal, and antiparasitic agents (48). The presence of compounds with these properties in the fish's system may block or inhibit the growth of pathogens, thus helping the immune system to eliminate disease-causing agents (48). Some compounds found in some plants such as carvacrol and thymol have been shown to improve gut health and intestinal immunity (50).

Many studies have shown that plants can successfully enhance some of the components of innate immunity in *O*. *mossambicus* and *C*. *gariepinus* and increase survival against common bacterial pathogens (Table 2). It is interesting that some of these plants have also been shown to improve growth in these fish species (Table 1), which imply that there is an excellent chance for these plants to be included in aquafeeds. For example, the addition of *Z*. *officinale* powder in the diets of *O*. *mossambicus* resulted in significant improvement in innate immune parameters, namely phagocytic activity, respiratory burst, lysozyme activity, total protein, and globulin (23). The authors also found an increase in survival rate after fish were challenged with *Vibrio vulnificus* (23). Similarly, Dügenci et al. (51) showed that the use of *Z*. *officinale* powder in the diets of the same species also enhanced immunity. As mentioned before, ginger is one of the most common plants with promising potential because it is widely available in many communities. Ginger has an advantage because it has been shown to improve growth performance in both *O*. *mossambicus* and *C*. *gariepinus* (23, 24). Furthermore, ginger has been reported to influence the health (hematological and biochemical) status in other fish species (52). Concerted efforts are required in Southern Africa to popularize its potential use in fish farming.

**Table 2:** Plants that have been shown to enhance immunity and disease resistance in *Oreochromis mossambicus* and *Clarias gariepinus*.

**Fish species**	**Plant species**	**Dose**	**Effect**	**Increased survival against**	**References**
*Oreochromis mossambicus*	*Zingiber officinale* (ginger)	1%	↑ Phagocytic activity, respiratory burst, lysozyme activity, total protein, and globulin	*Vibrio vulnificus*	(23)
	*Zingiber officinale*	0.1 and 1%	↑ Phagocytosis and extracellular burst activity of the blood leukocytes	N/A	(51)
	*Thymus vulgaris* (thyme)	1%	↑ Haematocrit, red blood cell, and innate immune response (plasma myeloperoxidase activity, lysozyme activity, phagocytic activity, white blood cell, neutrophil, and monocyte counts)	*Streptococcus iniae*	(53)
	*Rosmarinus officinalis* (rosemary)	1%	↑ Haematocrit, red blood cell, and innate immune response (plasma myeloperoxidase activity, lysozyme activity, phagocytic activity, white blood cell, neutrophil, and monocyte counts)	*S*. *iniae*	(53)
	*Trigonella foenum graecum* (fenugreek)	1%	↑ Haematocrit, red blood cell, and innate immune response (plasma myeloperoxidase activity, lysozyme activity, phagocytic activity, white blood cell, neutrophil, and monocyte counts)	*S*. *iniae*	(53)
	*Thymus vulgaris*	0 and 1.0%	N/A	*S*. *iniae*	(54)
	*Rosmarinus officinalis*	0 and 1.0%	N/A	*S*. *iniae*	(54)
	*Trigonella foenum graecum*	0 and 1.0%	N/A	*S*. *iniae*	(54)
	*Thymus vulgaris*	0 and 1.0%	N/A	*S*. *iniae*	(55)
	*Rosmarinus officinalis*	0 and 1.0%	N/A	*S*. *iniae*	(55)
	*Trigonella foenum graecum*	0 and 1.0%	N/A	*S*. *iniae*	(55)
	*Citrus sinesis* (sweet orange)	1, 3, 5 g kg^−1^	↑ Lysozyme, myeloperoxidase, total protein, and globulin	*S*. *iniae*	(57)
	*Citrus limon* (bitter lemon)	0.5, 0.75, 1%	↑ Respiratory burst, lysozyme, myeloperoxidase, and total protein	*Edwardsville tarda*	(58)
	*Tinospora cordifolia*	0.0008%	N/A	*Aeromonas hydrophila*	(59)
	*Tinospora cordifolia*	0, 6, 60, and 600 mg kg^−1^	↑ Antiprotease, complement, lysozyme activities, and reactive oxygen and nitrogen species	N/A	(60)
	*Ocimum sanctum*	0.0001, 0.01, and 1%	↑ Innate immune response	*A*. *hydrophila*	(62)
	*Cuminum cyminum*	0, 0.5, 1, 1.5, and 2.0%	↑ Innate immune response	*S*. *iniae*	(37)
	*Tribulus terrestris*	0, 200, 400, 600, and 800 mg kg^−1^	N/A	*S. iniae*	(40)
	*Pimenta dioica* (allspice)	10, 15, and 20 g kg^−1^	↑ Serum glucose, plasma lysozyme activity, myeloperoxidase activity, total protein, albumin, and globulin	N/A	(63)
	*Pimenta dioica*	5, 10, 15, and 20 g kg^−1^	N/A	*S. iniae*	(45)
	*Pimenta dioica*	0, 5, 10, 15, and 20 g kg^−1^	↑ Red blood cell, mean corpuscular volume, mean corpuscular hemoglobin, respiratory burst activity, lysozyme, and myeloperoxidase	N/A	(64)
	*Artemisia afra*	0, 3, 6, 9, and 12%	↑ White blood cells, lysozyme, and phagocytic activities	*A*. *hydrophila*	(65)
	*Moringa oleifera*	0, 3, 6, 9, and 12%	White blood cells, lysozyme, and phagocytic activities	*A*. *hydrophila*	(69)
*Clarias gariepinus*	*Artemisia afra*	0, 3, 6, 9, and 12%	↑ White blood cells, lysozyme, and phagocytic activities	*A*. *hydrophila*	(66)
	*Moringa oleifera*	0, 3, 6, 9, and 12%	↑ White blood cells, lysozyme, and phagocytic activities	*A*. *hydrophila*	(70)
	*Aloe vera*	0, 0.5, 1.0, 2.0, and 4.0%	↑ Haemato-biochemical parameters (Red blood cells, hematocrits, hemoglobin, platelets, White blood cells, lymphocytes, monocytes, and granulocytes)	N/A	(30)

The symbol ↑ represents an increase in all parameters. N/A, Not applicable.

Dietary effects of thyme (*Thymus vulgaris*), *R*. *officinalis* and *Trigonella foenum graecum* (fenugreek) leaf powder increased hematology (haematocrit, red blood cell) and innate immune response (plasma myeloperoxidase activity, lysozyme activity, phagocytic activity, white blood cell, neutrophil, and monocyte counts) in *Oreochromis mossambicus* (53). Furthermore, fish fed with the diets containing these plants displayed increased resistance when challenged with *Streptococcus iniae* (53). Gurkan et al. (54) also reported that diets enriched with *T*. *vulgaris, R*. *officinalis*, and *T*. *foenum graecum* enhanced resistance in *O*. *mossambicus* against *S*. *iniae*. The findings from these studies are consistent with Yilmaz's et al. (55) results, which showed that thyme, rosemary, and fenugreek leaf powder significantly lowered mortality rates in *O*. *mossambicus* fry infected with *S*. *iniae*. These studies concluded that these plants can be used as immunostimulants to improve immune response and disease resistance against *S*. *iniae* in *O*. *mossambicus*. Among these plants, *R*. *officinalis* is the most commonly researched plant and it has been shown to improve growth performance in *C*. *gariepinus*. *Rosmarinus officinalis* is commonly used in traditional medicine because it is a rich source of bioactive compounds (55). *Thymus vulgaris* is a Mediterranean herb known for its dietary, medicinal, and ornamental uses. There is a significant potential for its use in aquaculture in Southern Africa because various products from thyme are now available in many countries. Fenugreek is a plant occurring in Northern Africa, the Mediterranean, western Asia, and Northern India. It is mainly used medicinally and for food purposes (as a spice and vegetable). This plant is now being cultivated in many countries and fish farmers in Southern Africa can benefit from its use. There is thus an opportunity for farmers in Southern Africa to consider using these plants as alternative feed additives to increase immunity in tilapia culture.

*Citrus sinesis* (sweet orange) is another plant with a significant potential to be used to boost immunity in fish. It is commonly cultivated in Southern Africa and it is reported to contain compounds with significant medicinal value. For instance, it is known to contain vitamin C, which is a strong antioxidant (56). Acar et al. (57) showed that the inclusion of *C. sinesis* oil in *O*. *mossambicus* diets improved lysozyme, myeloperoxidase, total protein and globulin. Acar et al. (57) also found that the diets increased resistance when *O*. *mossambicus* were infected with *S*. *iniae*. Baba et al. (58) found that enriching diets with *C*. *limon* (bitter lemon) enhanced innate immunological parameters (respiratory burst, lysozyme, myeloperoxidase, and total protein) and conferred resistance against *Edwardsville tarda*. *Citrus limon* also occurs abundantly in Southern Africa where it is cultivated for medicinal purposes. The lack of its use in aquaculture shows that farmers may be lacking knowledge about its potential use as an immunostimulant in fish. Sudhakaran et al. (59) showed that immunity was improved in *O*. *mossambicus* fed with a diet containing *Tinospora cordifolia* Miers leaf extract and survival rate increased when the fish were exposed to *A*. *hydrophila*. Alexander et al. (60) also confirmed this when they showed that immune parameters (i.e., antiprotease and complement, activities lysozyme, reactive oxygen, and nitrogen species) increased in *O*. *mossambicus* after feeding with diets supplemented with *T*. *cordifolia*. *Tinospora cordifolia* is indigenous to the Indian subcontinent where it is used in herbal medicine (61). Its distribution outside of India seems very limited and therefore it cannot be used widely in aquaculture on the African continent. Logambal et al. (62) reported that feeding *O*. *mossambicus* with diets enriched with *Ocimum sanctum* increased immunity and offered protection against *A*. *hydrophila*. Results obtained from a study conducted by Yilmaz et al. (37) showed that diets supplemented with *C*. *cyminum* seed meal elevated the innate immune response in *O*. *mossambicus* and increased survival rate after a challenge with *S*. *iniae*. This plant also improved feed utilization in the same fish (37), making it one of the plants of interest in freshwater. Its use in aquaculture should be highly encouraged especially because it is now available worldwide. Dietary supplementation with *Tribulus terrestris* extract enhanced disease resistance in *O*. *mossambicus* and protected against *S*. *iniae* infection (40). *Tribulus terrestris* also increased performance in *O*. *mossambicus* (40). *Pimenta dioica* powder supplementation has also been reported to influence some immunological and serum biochemical status (i.e., serum glucose, plasma lysozyme activity and myeloperoxidase activity, total protein, albumin, and globulin) in *O*. *mossambicus* (63). Yilmaz and Ergün (45) also reported that the use of *P*. *dioica* had a positive effect on disease resistance in *O*. *mossambicus*, which was demonstrated by an increase in survival rate after a challenge with *S*. *iniae*. Another study conducted by Yilmaz et al. (64) also found that diets supplemented with allspice improved hematological indices (red blood cell, mean corpuscular volume, mean corpuscular hemoglobin) and some innate immune parameters such as respiratory burst activity, lysozyme, and myeloperoxidase activities in the same fish species. Growth performance also improved in *O*. *mossambicus* fed with diets supplemented with *P*. *dioica* powder (45). This is an indication that its use in aquaculture should be encouraged.

There are also plants that are currently widely used in traditional medicine in Southern Africa, which have potential to be used as immunostimulants in *O*. *mossambicus* and *C*. *gariepinus*. Investigations undertaken by Mbokane and Moyo (65) found that *A*. *afra* leaf powder increased innate components such as white blood cells, respiratory burst, and lysozyme activity in *O*. *mossambicus*. Their results also showed that *O*. *mossambicus* fed with the *A*. *afra*-based diets had increased resistance against *A*. *hydrophila*. Mbokane and Moyo (66) also showed that the use of *A*. *afra* leaf powder in the diets of *C*. *gariepinus* improved immunity (WBCs, respiratory burst, and lysozyme activity) and increased resistance against *A*. *hydrophila*. *Artemisia afra* is one of the most popular medicinal plants used in traditional medicine in Southern Africa (67, 68). It is clear that *A*. *afra* contains compounds that can improve immunity in these fish species and thus its use in aquafeeds should be promoted among fish farmers.

*Moringa oleifera* is another popular and widely available plant in Southern Africa that has been shown to possess immunostimulatory properties. Although Hlophe-Ginindza and Moyo (17) reported that moringa's inclusion in the diets of *O*. *mossambicus* and *C*. *gariepinus* led to poor growth rates and affected fish health, they noticed that white blood cells increased with increasing moringa levels in the diet. This was probably an indication that it could be used as an immunostimulant as opposed to a growth-promoter. Mbokane and Moyo (69) confirmed this when they found that diets supplemented with *M*. *oleifera* leaf powder (3–12%) enhanced innate immune response (WBCs, respiratory burst, and lysozyme activity) in *O*. *mossambicus*. They also reported that fish fed with diets supplemented with moringa had increased resistance against *A*. *hydrophila*. Similarly, Mbokane and Moyo (70) showed that the moringa-based diets improved innate immunity and survival increased in *C*. *gariepinus* after a challenge with *A*. *hydrophila*. This indicates that moringa can be used as an immunostimulant when used as a supplementation (feed additive) as opposed to replacing fishmeal in fish diets. The other commonly occurring plant in Southern Africa that has been reported to enhance immunity in fish is *A*. *vera*. This plant has been reported to increase several immune parameters (white blood cells, lymphocytes, monocytes, granulocytes) in *C*. *gariepinus* (30). It was also found to have enhanced growth performance in the same fish species, which demonstrates its potential benefit as one of the plants that can be used in fish diets.

Overall, the literature shows that there are several common and locally available plants that can be used as alternatives to antibiotics and synthetic chemicals in controlling bacterial diseases in tilapia and catfish culture. Although no studies used a fungal pathogen as a test organism, the fact that these plants have been shown to increase immune capacity imply that the fish can resist various pathogens. As mentioned in the outset, diseases in Southern Africa are mainly caused by bacterial and fungal pathogens, which means that increased resistance to these pathogens can improve production. This shows that the application of medicinal plants can play a central role in preventing pathogenic diseases in the freshwater aquaculture sector in Southern Africa.

## Use of plants to improve reproduction

Plants can also be used to control or enhance reproduction in fish. It has been shown that some compounds found in plants could control reproduction in tilapia (12). Plants contain androgenic compounds, which have been reported to disrupt the reproductive cycle in fish (71–74). Phytoandrogens such as testosterone, androstenedione and dehydroepiandrosterone have been shown to reverse the sex of fish (74), which can thus be used to reduce unwanted reproduction in tilapia. Most of the phytochemicals (i.e., such as steroids, saponins, flavonoids, alkaloids, tannins) found in plants inhibit enzymes required for the production of sex hormones (74). This is known to block the synthesis and activities of testosterone or estrogens (12). For example, the inhibition of estrogen synthesis can reduce fertility, which could be a suitable option to control reproduction in tilapia (74). In tilapia culture, one of the interventions needed to increase production is the elimination of prolific breeding, resulting in overpopulation with stunted and small-sized fish, which are not good for the market. Generally, tilapia farmers prefer to culture all-males to avoid inbreeding and because males grow faster than females. It may thus benefit tilapia farmers in Southern Africa to consider the use of plant additives to control or change the sex of the fish in order to enhance production.

Although no plants have specifically been tested on *O*. *mossambicus*, several plants have been reported to control reproduction in other tilapia species such as *O*. *niloticus* (Nile tilapia). These include, among others, *G*. *kola, Gossypium herbaceum* (Cotton), *T*. *foenum graecum, Psidium guajava* (Guava), *Mangifera indica* (Mango), *M. oleifera, Carica papaya* (Pawpaw), *Pinus* spp. (Pine trees), and *A*. *vera* (74). All these plants are readily available in Southern Africa and some have also been reported to enhance growth and immunity in some fish species. On the other hand, some studies reported that the inclusion of medicinal plants in the diets of *C*. *gariepinus* can promote sexual maturation (11, 75, 76). Mehrim et al. (11) recently evaluated the effect of Ginseng^®^ on reproductive development in *C*. *gariepinus*. Their results showed that 200 mg Ge/kg diet significantly increased gonado-somatic index, serum follicle-stimulating hormone, sperm quality, and ultrastructure of spermatozoa. Ginseng^®^ is a commercial plant product that can be easily accessible to fish farmers. Dada and Adeparusi (75) investigated the use of dietary effects of two medicinal plants, namely *Sesamum indicum* (Sesame) and *Croton zambesicus* seed powder on the reproductive indices in female African catfish broodstock. They found that both plants increased gonadosomatic index and reproductive indices in the female broodstock. Sesame is a traditional oilseed mainly grown in Asia and Africa, where it is used in folk medicine (77). *Croton zambesicus* is a medicinal also used in folk medicine in Africa. *Garcinia kola* has also been reported to improve sperm and egg quality in *C*. *gariepinus* (78–80). Dietary supplementation with *Z*. *officinale* dried powder also increased sperm motility, sperm life span, and egg weight in *C*. *gariepinus* (25). These plants can play a significant role in freshwater aquaculture in Southern Africa as they also improved growth performance in the same species (25, 38).

*Kigelia africana* (Lam. Benth) dried fruit meal is another plant that has been shown to enhance fertility (ovulation and spermiation) in *C*. *gariepinus* (81, 82). This plant grows abundantly throughout tropical Africa where it is widely used as a herbal remedy for various human ailments (i.e., dizziness, retained placenta, rheumatism, diarrhea, and malaria) (83). It is also used treat sexual health problems such as poor libido, impotence, infertility, and sexual asthenia (84). Furthermore, this plant has been experimentally shown to enhance fertility in rats (85). This is an indication that its usage can enhance fertility in aquaculture. The use of *Desmodium adscendens* (desmodium) leaf powder also led to an increase in sperm count and milt volume in *C*. *gariepinus* males while the weight of ovaries increased in *C*. *gariepinus* females (76). However, *D*. *adscendens* grows abundantly in the equatorial regions of Africa and may not be readily available in Southern Africa.

The mechanism involved in the enhancement of fertility in fish at molecular level is not clearly understood. However, most of the plants shown to increase fertility in fish possess bioactive compounds known to improve overall wellbeing in fish through detoxification and by reducing oxidative stress by scavenging free radicals. This is known to regulate and improve various essential body functions (76). Some plants have been shown to contain compounds that provides protection against lipid peroxidation. For example, Ofusori et al. (86) demonstrated that sperm production and sperm motility improved in swiss albino mice after treatment with leaf extract of *C*. *zambesicus* supplemented diets. They also found that a significant reduction in Malondialdehyde (MDA) concentration in the testes of treated groups. They concluded that *C*. *zambesicus*' antioxidant activity in the testes may have inhibited lipid peroxidation and thus increased fertility in the mice. It is thus reasonable to infer that plants may have a similar effect on reproductive organs in fish. It is therefore recommendable that farmers in Southern Africa seriously consider the application of medicinal plants in tilapia and catfish in an effort to improve production.

## Main perspective

This review shows that several plants can be used as feed additives in aquafeeds in Southern Africa in order to enhance growth performance, disease resistance, and reproduction in *O*. *mossambicus* and *C*. *gariepinus*. Indigenous and common plants such as *A*. *afra, M*. *oleifera, A. sativum, A*. *vera, Z*. *officinale, C*. *limon*, and *C*. *sinesis* have the greatest potential to be used as feed additives in aquafeeds in Southern Africa. These plant species are widely available in Southern Africa and can be cultivated in backyards. Some of their products have been commercialized and are available in many countries. In Southern Africa, plants such as *A*. *afra, M*. *oleifera*, and *A*. *ferox* have been partially or fully processed as commercial products and are now available in various dosage forms such as tablets, capsules, and teas (87). However, there is limited research in Southern Africa focusing on the utilization of medicinal plants in freshwater aquaculture. Therefore, more research should focus on the local pharmacopeia in order to assess its potential as feed additives or immunostimulants. Southern Africa boast a diverse number of medicinal plants, which is reported to account for over 10% of the world's medicinal plant species (87). However, only few indigenous plant species have thus far been investigated for their inclusion in aquafeeds. Majority of the medicinal plants found in Sothern Africa contain many bioactive compounds such as volatile oils, flavonoids, polyphenols, alkaloids, polysaccharides, organic acids, saponins, and tannins. They also contain minerals, vitamins carbohydrates, and amino acids. These compounds make these plants suitable candidates for possible use as feed additives in aquafeeds.

The use of plants in aquafeeds can benefit small-scale fish farmers the most, especially where prices for commercial diets, synthetic hormones, and antimicrobials are too expensive or access is limited. Small-scale fish farmers often lack resources and the application of plants can help them to reduce operational costs. Evidence from other countries has shown that the utilization of medicinal plants can help rural farmers to increase production by promoting growth performance and minimizing incidences of diseases. For example, Caruso et al. (88) found that 46% of fish farmers in rural areas in Indonesia used plants on their farms to treat fish diseases and to improve the capacity of fish's immune response against pathogens. The authors reported that most of the plants used on the farms were indigenous plants used in traditional medicine. Furthermore, the application of plants in aquaculture is already being practiced in Bangladesh (89), China (90), and other countries (9). This highlights the important role that indigenous plants can play in aquaculture. Therefore, the utilization of medicinal plants in freshwater aquaculture especially in the small-scale sector in Southern Africa can be an opportunity to use alternative and affordable feed additives as protein sources and immunostimulants. Farmers should be encouraged to utilize some of the tested plants when formulating fish diets. Diets supplemented with plant products can be easily produced on farms without the need for sophisticated technology or more resources. All that is required is dissemination of information among farmers about the beneficial effect plants may have on cultured fish.

## Conclusions and recommendations

Currently, there are some limitations concerning the wide application of plants in aquafeeds in Southern Africa. This may be attributed to the fact that there are very few studies that have been conducted at farm level. In the future, studies conducted on indigenous and common plants should focus on their effect at production level in order to popularize their use among farmers. At present, much of what is available regarding the use of plants as feed additives is still at research level. Thus, it is critical to intensify efforts looking at increasing the use of plant-based in freshwater aquaculture. This review shows that plants can be used as feed additives to improve fish production. We strongly recommend the utilization of plants as feed additives especially in the small-scale sector where production is hampered by poor quality of feeds, precocious breeding, poor condition of breeders, and disease outbreaks. Efforts are also required to improve the dissemination of information on the application of medicinal plants in aquaculture among fish farmers. Many farmers are not aware that plants can be used to address some of the challenges they face on their farms. This is concerning because majority of fish farmers are aware that some medicinal plants are used in traditional medicine, but they do not know that they can also be used in fish. This gap in knowledge appears to be the major bottleneck that hinders the successful use of medicinal plants in the freshwater aquaculture industry in Southern Africa. As a result, there is a need for governments, academia, and research institutes to set up educational programs to train small-scale fish farmers about the potential of using medicinal plants in fish farming.

## Author contributions

EM and NM conceived the study, reviewed the literature, and drafted the manuscript. All authors contributed to the article and approved the submitted version.
